# Daily Star and Herald (Panama)

**Published:** 1884-05-20

**Authors:** 


### *DAILY STAR AND HERALD (PANAMA.)*

Through the kindness of a medical friend. we have had the pleasure of perusing the above paper, published in Panama. The Isthmus of Panama has been made prominent of late by reason of the great Inter Oceanic Canal, the work upon which is now in progress, with the famous de Lesseps as the manager.

Among many interesting items in the number before us is a paper relating to sanitary matters, in which the writer graphically protests against the almost utter disregard of sanitary regulations in this part of the world, which is to be called the Gate to the Pacific, at a point where yellow fever may be regarded as a perpetual fixture, if not indigenous to the locality. As an instance of the disregard or indifference to the laws of decency and health which prevails, we copy the following extract from the paper referred to:

"Of the two native cemeteries, one consists of a quadrangle of vaults or niches. The other is the generel burial ground. We consider the latter first. It is used by the population at large, as well as for the interment of paupers, prisoners and hospital patients. It faces the main road, and consists of three-fourths of an acre. This cemetery never was adequate to its duty. Panama to-day has a population of, say twenty thousand souls, constantly increasing. The cemetery lies within half a mile of the western suburbs of the city. Increase of population of necessity means more deaths, still this three-fourths of an acre *must do duty still.* When one tries to realize the fact that it is dug up year after year, and that its ghastly contents are turned to the broad light of day, one can then, and then only, estimate what a pestilence disease producing centre it becomes. How are its dead buried? Fully one-half without coffins. Coffins are secured at a small rental for the funeral; at the grave the dead are taken out, wrapped in a sheet and buried. The coffin is returned to the shop of the undertaker, in its turn to serve as a disease-producing factor. Owing to the smallness of the lot, in twelve months or less the coffins, or bones, or both, as the case may be, are rudely disturbed to make way for 'new arrivals' and are cast out of their temporary homes. Within that lot one sees scattered about coffins, pieces of coffins, bones, skulls, parts of clothing, etc., etc. For decency's sake, they are gathered together occasionally and burned, but what of the germs? This goes on under our intertropical sun, with an average yearly temperature of 84 deg. in the shade. Can health be expected under such circumstances? These germs in millions are cast loose from what should be their final resting place, and fly abroad to cause new disease and death, as shown by Dr. Domingo Freire, of Rio de Janeiro, and thus "perpetuate their species." This is no sensational pen and ink sketch; but a statement of things as they exist at this very time, as we write. This dreadful burying and unburying goes on from year to year, in strict conformity with the "colonial tradition," as many know, and truthful people will candidly admit."

